# Continuous periprosthetic bone loss around the TOP^®^ cup and inferior survival rate at an 8-year follow-up: a prospective cohort study

**DOI:** 10.1186/s12891-024-07865-5

**Published:** 2024-09-16

**Authors:** Demostenis Kiritopoulos, Andreas Nyström, Nils P. Hailer, Hans Mallmin, Stergios Lazarinis

**Affiliations:** Department of Surgical Sciences, Section of Orthopedics, Uppsala University, Uppsala, Sweden

**Keywords:** Uncemented THA, Cup, DXA, Prospective study, TOP cup

## Abstract

**Background:**

The trabeculae-oriented pattern (TOP^®^) cup was designed to minimize acetabular periprosthetic bone loss. In our previous prospective study comprising 30 patients with a two-year follow-up we found a substantial decrease in periprosthetic bone mineral density (pBMD) in the proximal and medial regions of the TOP cup. The present study aims to investigate pBMD changes in the mid-term and how this affects implant survival.

**Methods:**

We followed the previous cohort and estimated implant survival by Kaplan-Meier analysis, evaluated pBMD with dual-energy X-ray absorptiometry (DXA) and clinical outcome using the Harris Hip Score (HHS).

**Results:**

Mean follow-up was 8.6 (range 7.8–9.1) years. The eight-year implant survival rate for cup revision for all reasons was 83% (95% confidence interval {CI}: 70–97) and 86% (CI: 74–99) when cup revision due to aseptic loosening was the endpoint. Mean HHS at eight years was 95 (range 77–100). A further 12% (CI: 5–17) loss in pBMD was detected in the proximal Digas zone 1 and 12% (CI: 7–17) loss in Digas zone 2 also between two and eight years after surgery. pBMD continued to decrease up to 30% (CI: 24–36) in Digas zones 1, 2 and 3 compared to pBMD immediately postoperatively.

**Conclusions:**

The TOP cup shows inferior mid-term survival rates compared to other uncemented cups, as well as a continuous decrease in pBMD. Periprosthetic bone loss cannot be prevented by this uncemented cup.

**Clinical trial number:**

Not applicable.

**Supplementary Information:**

The online version contains supplementary material available at 10.1186/s12891-024-07865-5.

## Background

Total hip arthroplasty (THA) is one of the most commonly performed operations in orthopedic surgery. Although implant survival and clinical outcomes in most cases range from good to excellent, the need for revision surgery remains in about 10% of patients within a 10-year perspective [1]. The most common cause of revision arthroplasty is aseptic acetabular component loosening. During the first year after uncemented THA, periprosthetic bone mineral density (pBMD) loss has often been reported [2, 3]. pBMD loss at the acetabulum has been suggested to be mediated by several mechanisms, including “backside wear”, stress shielding, polyethylene (PE) wear and a poor locking PE mechanism [4–6]. The trabeculae-oriented pattern (TOP^®^) cup is a press-fit cup developed to minimize acetabular periprosthetic bone loss with a segmental row of threads around the equator to ensure stable anchorage in the acetabulum. The TOP cup is designed with a mediocaudal recess to provide a wider range of motion in adduction and prevent impingement from the neck of the stem.

In the original study on the TOP cup at our institution [7] we reported a reduction of BMD in the proximal and medial regions (Digas zones 1, 2 and 3) two years after surgery. The current study extended the follow-up of the TOP cup by investigating eight-year implant survival and pBMD as measured by dual-energy X-ray absorptiometry (DXA). Clinical results were assessed with the Harris Hip Score (HHS).

## Methods

### Study design and population

30 patients with primary osteoarthritis of the hip eligible for uncemented THA were included in the original study [7]. Inclusion criteria were symptomatic and radiographically verified osteoarthritis of the hip, age 20–65 years, and a body weight below 100 kg. Exclusion criteria were inflammatory diseases (rheumatoid arthritis and comparable diseases) as diagnosed by the American Rheumatism Association (ARA) criteria, systemic glucocorticoid treatment, disabling diseases from the musculoskeletal system other than the hip, malignancy, alcohol or drug addiction, psychiatric disorders, chronic infectious diseases, and osteoporosis (as determined by DXA). Further exclusion criteria were severe joint deformities unsuitable for the use of the collum femoris-preserving (CFP) stem. One patient was not eligible for further follow-up because of a revision caused by an early deep infection. Thus, the final cohort included 29 patients with primary hip osteoarthritis.

In the present study all 29 patients were invited to participate in a follow-up (on average 8.6 years after surgery) investigation with clinical examination, HHS, radiology and pBMD measurement with DXA. Exclusion criteria for the clinical follow-up were cup revision surgery or death, whichever occurred first.

### Implant and surgery

All patients received the uncemented TOP cup (Waldemar Link GmbH & Co.KG, Hamburg, Germany) and the uncemented CFP stem with a 28 mm metal head (Waldemar Link GmbH & Co.KG, Hamburg, Germany).

**Fig. 1:**
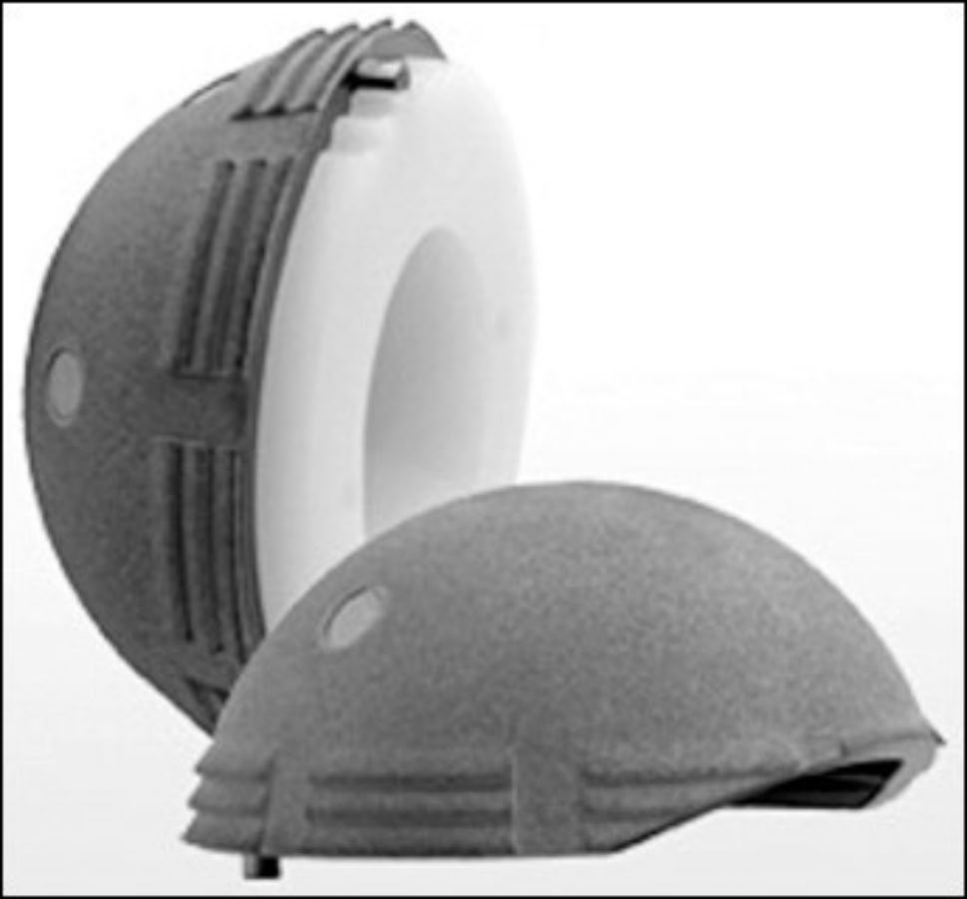
The Trabeculae Oriented Pattern (TOP) cup

The TOP cup is a press-fit hemispherical cup made of titanium alloy (Tilastan^®^) with a 70 μm microporous surface and a 15 μm thick hydroxyapatite (HA) coating (Fig. 1). The inserts are constructed of ultra-high-molecular-weight polyethylene (UHMWPE), sterilized but not highly “cross-linked”. Two experienced surgeons performed all the operations. Details of the surgical procedure and implants are available in the original study [7].

### Dual-energy X-ray absorptiometry (DXA)

DXA scans of the operated hip were performed with the same system as in the original study: Prodigy Advance System (GE-Lunar, Madison, WI, USA). The pBMD in the five zones was analyzed according to Digas [8]. The precision of the periprosthetic acetabular DXA measurements in our institution with repeated scans after repositioning, expressed as a coefficient of variation (CoV), has been previously described [7].

### Clinical outcome measures and radiology

The HHS was used to assess patient-reported outcomes of hip function. Anteroposterior and lateral radiographs of the hip and a standard pelvic view were obtained. Two examinators evaluated the X-rays to determine signs of cup migration or loosening.

### Statistics

Sample size calculations have been described in the original study [7]. All variables were summarized using standard descriptive statistics. The within-subjects effect was calculated using repeated measures ANOVA. The assumption of sphericity was determined by Mauchly’s test. If sphericity was violated, the Greenhouse-Geisser correction was applied. The significance level was set at 0.05 and 95% confidence intervals (CIs) were calculated. Implant survival was estimated using Kaplan-Meier (KM) analysis. All statistical calculations were performed using SPSS Statistics version 27 (IBM Corp, Armonk, NY, USA).

### Ethics, funding and potential conflict of interests

All patients gave oral and written consent to participate. The study was approved by the Regional Ethics Committee in Uppsala (Dnr 2007/105) and complied with the Declaration of Helsinki. NPH has received institutional grants and personal fees as lecturer from Waldemar Link GmbH, Heraeus, and Zimmer Biomet. SL has received institutional grants as lecturer from Waldemar Link GmbH and Heraeus. The other authors reported no conflicts of interest related to the study.

## Results

### Characteristics of the study population

**Fig. 2:**
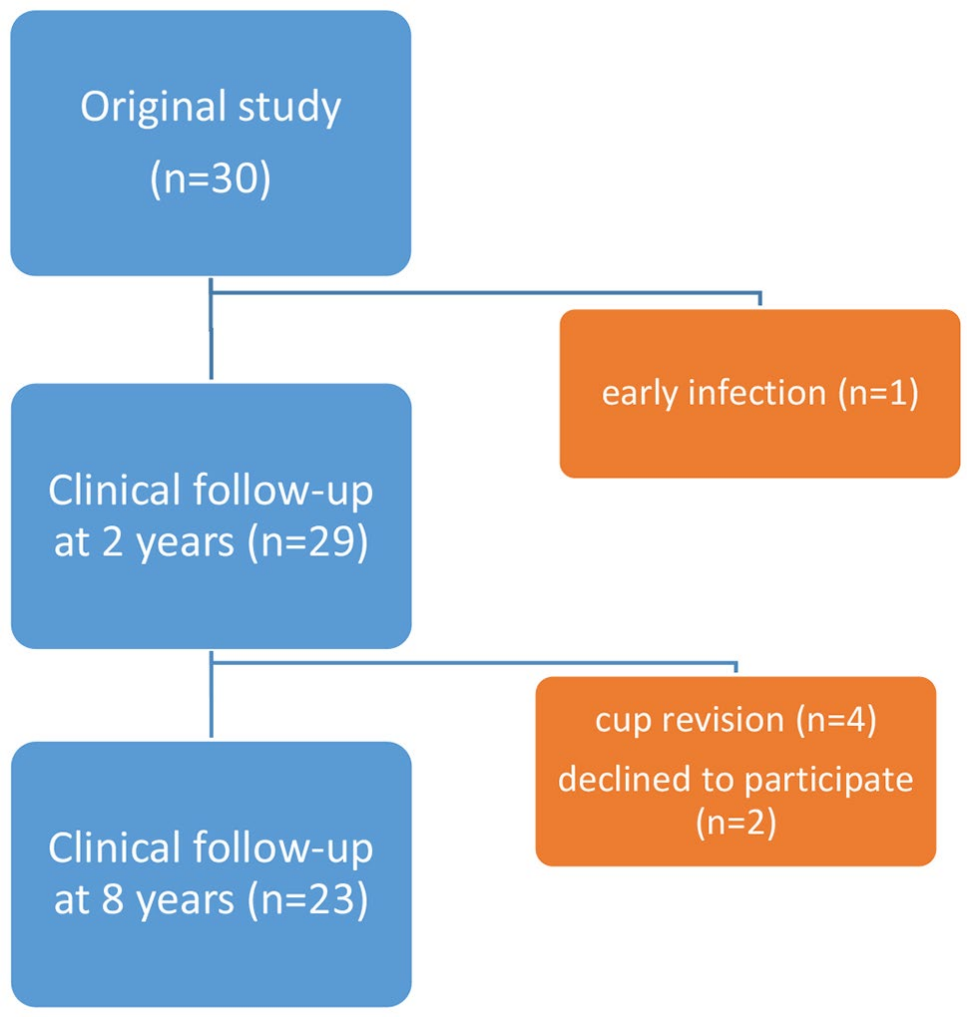
Flowchart of eight-year clinical follow-up

30 patients (18 women) with a mean age of 56 (range 42–65) years at the time of index surgery, were recruited from March 2008 to March 2009 and included in the original study. Five patients underwent revision surgery of the cup and were therefore not eligible for clinical follow-up. Two patients refused to participate but reported no implant-related problems after telephone contact (Fig. 2). Thus, 23 patients (12 women) with a mean age of 64 (range 55–73) years and a median BMI of 26 (20–31) participated in the clinical follow-up.

### Implant survival and revision

As reported in the original study, one patient underwent revision because of an early deep infection three months after surgery. Three patients had undergone isolated cup revision and one total hip revision (both cup and stem) during two to eight years of follow-up. Thus, we found an eight-year cup survival rate for all reasons of 83% (CI: 70–97). When revision due to aseptic loosening of the cup was the endpoint in the KM analysis, the implant survival rate was 86% (CI: 74–99).

A review of patients’ surgery journals revealed macroscopically significant PE liner wear in two of four patients who had undergone revision due to cup loosening.

### Dual-energy X-ray absorptiometry

**Fig. 3:**
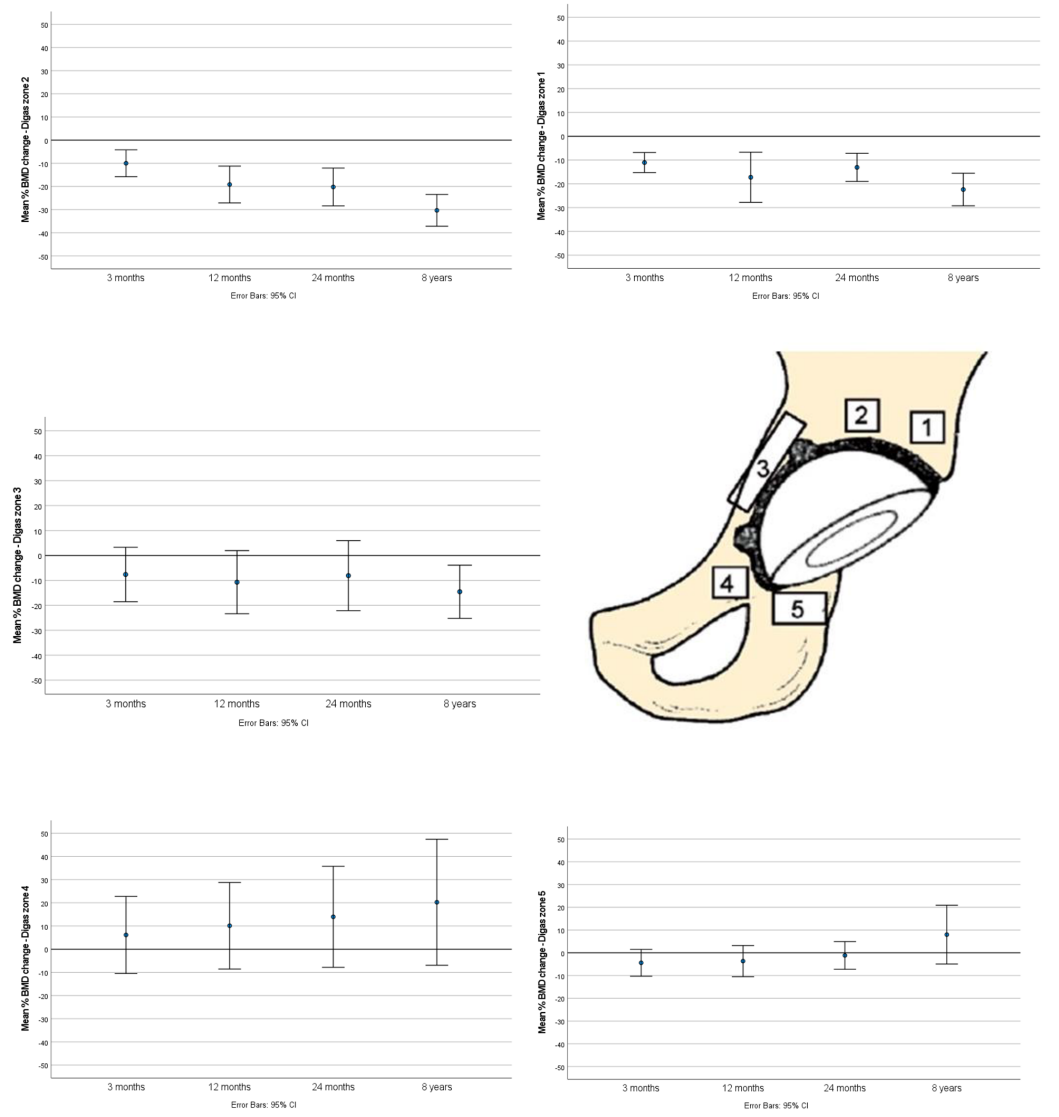
Changes in periprosthetic bone mineral density from baseline in Digas zones 1–5 (*n* = 21). Error bars represent 95% confidence intervals

We found a continuous decrease of 12% (CI: 5–17) of pBMD in the proximal periprosthetic Digas zone 1 (*p* = 0.005) and a reduction of 12% (CI: 7–17) in Digas zone 2 (*p* = 0.003) two to eight years after surgery. A smaller, statistically non-significant decrease in pBMD (5%, CI: 2–12) was also seen in Digas zone 3 (medially). In Digas zone 4 (ramus superior) and 5 (the ischial tuberosity) continuous non-significant increases in pBMD of 8% (CI: -7-21) and 10% (CI: 2–18), respectively, were recorded (Fig. 3).

Compared to the baseline (direct postoperatively), we found a 22% (CI: 16–28) reduction (*p* < 0.001) in pBMD in Digas zone 1, a 30% (CI: 24–36) reduction (*p* < 0.001) in Digas zone 2 and a 15% (CI: 5–24) reduction (*p* = 0.01) in Digas zone 3. A non-statistically significant increase in pBMD was observed in Digas zone 4 (15% CI: -7-37, *p* = 0.5) and 5 (8%, CI: -4-20, *p* = 0.6).

### Clinical outcome measures and radiography

Clinical outcome at the eight-year follow-up was excellent, with a mean HHS of 95 (77–100). 22 of 23 plain digital anterior-posterior radiographs of the pelvis were assessable. No signs of cup loosening were observed in any patient, but migration of the hip head was seen in eight hips.

## Discussion

The original study found excellent short-term results and good primary stability but pBMD loss was found in the proximal zones around the TOP cup. We report two key eight-year results for the TOP cup in the present mid-term follow-up study. First, an inferior cup survival rate was noted compared to other uncemented cups, and second, pBMD continued to decrease in the proximal zones.

### Implant survival

In the original study, one patient required revision surgery because of early deep infection, and there were no further cup revisions presenting an excellent cup survival rate of 97% at two years. In this prospective study following 29 patients for eight years, four had undergone cup revision surgery because of aseptic loosening, reducing the overall survival rate of the cup to 83%. When the endpoint was aseptic loosening of the cup, the survival rate was reduced from 100% at two years to 86% at eight years. Our results are in in line with the reports on cup survival in the Swedish Arthroplasty Register for the TOP cup when used in combination either with the cemented Lubinus stem or with the CFP stem [9]. Two reports on the TOP cup indicate excellent performance with 100% survivorship after seven years [10, 11]. A recent retrospective study by Mosconi et al. of 662 TOP cups with a mean follow-up of 12 years showed a total survival rate of 91% [12]. The main reason for revision was aseptic loosening (39/50 cases, isolated or combined with stem loosening), followed by periprosthetic joint infections (7/50), PE wear (2/50) and dislocations (2/50). Despite a much longer follow-up, a possible explanation of this favorable survival rate compared to our results could be the use of a ceramic head and a UHMWPE liner and the next-generation highly “cross-linked” PE liner in their cohort. In contrast, we used a metal head and only the UHMWPE liner in our cohort. Using the metal head combined with non-“cross-linked” PE can lead to substantial liner wear, confirmed in two of the four cases revised in our study and subsequently leading to aseptic cup loosening. Additionally, in Mosconi’s study a considerable number (22%) of patients were lost to follow-up, which introduces an uncertainty of reported survival rates, including the possibility that worse results might be suspected in dropouts compared to follow-up participants. The aseptic loosening seen in the four patients in our study, could be interpreted as a late detection of loosening and not a genuine late onset of loosening.

Our assumption that the poor PE liner quality can lead to substantial liner wear, periprosthetic osteolysis and subsequently to aseptic cup loosening is challenged by the theory of early loosening [13]. According to this theory, implant loosening has already been initiated during or shortly after surgery due to poor interlock between the bone and the implant or because of poor bone quality. Micromovements of the implant cause high fluid pressure between the bone and the implant, inducing periprosthetic osteolysis by a series of inflammatory responses.

Mid-term results from retrospective, single-center, multicenter and register studies for various uncemented acetabular implants showed more than a 94% survival rate after > six years of follow-up [14–16]. The 15 μm thick HA coating around the TOP cup was thought to enhance bone ingrowth, stimulate bony gap filling and improve additional secondary implant stability. However, this type of coating does not prevent demineralization around various uncemented cups and can lead to third-body wear and negative effects on the long-term stability of the cup [3].

In summary, the mid-term survival rate for the TOP cup in this prospective study was inferior to retrospective studies with the same implant [10–12] and inferior compared to the survival rates from other uncemented acetabular implants [14–16].

### Periprosthetic BMD

The original study reported a substantial decrease of pBMD in the proximal zones around the TOP cup after two years. In the present study we found a mid-term steady reduction of pBMD in the proximal zones around the implant. These results align with other studies on pBMD in uncemented cups [17–19]. A similar pBMD loss was observed in the proximal zones around the uncemented Continuum^®^-cup in a previous study in our institution [18] and a prospective study on the uncemented Trilogy^®^-cup by Digas et al. [2], where the pBMD was measured with DXA. Two prospective studies with uncemented cups agree with our results, reporting a persistent decrease of pBMD two and five years after surgery [19, 20]. Conversely, Field et al. noted the preservation of pBMD in the proximal zones two years post-surgery [21].

The original study showed that the majority of the cups migrated along the y-axis, which is the most sensitive axis for detecting migration. Thus, one possible mechanism for the periprosthetic demineralization is the theory of early loosening meaning that slightly loose cups or loose PE liners (inserts), via periprosthetic fluid pressure fluctuations, cause necrotic osteocytes, which in turn release damage associated molecular patterns that reinforce the osteoclast activity [13, 22].

According to Wolff’s law, a pBMD increase in the proximal zones could be expected because of increased load and bone remodeling. Contrarily, studies have shown that rigid fixation of a press-fit cup transmits forces sideways to the periphery [23, 24], leading to stress shielding and causing bone loss around the uncemented cups. Although the clinical implication of proximal pBMD loss remains unclear, the proximal zones around the uncemented cups are the most common location for osteolytic lesions and one of the main reasons for revision surgery after THA [25].

### Clinical outcome measures and radiography

The original study’s clinical outcome was excellent, reporting an HHS of 99. Our follow-up study also reports excellent but slightly less favorable clinical results (HHS = 95), which is expected in this mid-term eight-year post-operative follow-up. That difference did not exceed the minimal clinically important difference [26] and is in line with other mid-term studies on the TOP cup that express an HHS of 87–96 [10–12].

**Fig. 4:**
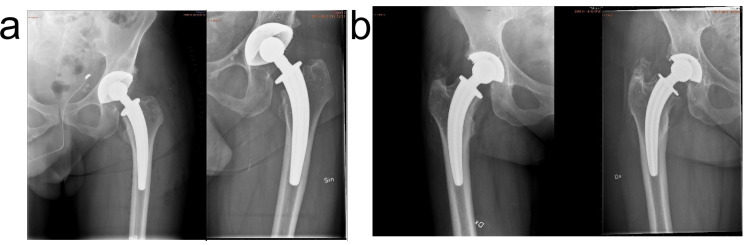
**a**. Radiography of a stable cup at eight years (right). Direct postoperatively image to the left. **b**. Radiography of a cup with PE wear at eight years (right.) Direct postoperatively image to the left

In the original two-year follow-up study no signs of cup or head migration were visible on X-rays. In our cross-sectional analysis of the anteroposterior pelvic radiography in this eight-year follow-up we observed a clear head migration in eight hips, indicating PE liner wear (Fig. 4a and b). A critical factor in the development of periprosthetic osteolysis is the biological reaction to wear debris from the PE liner. This reaction partly depends on the size and concentration of the particles, determined by the PE wear rate [27]. In two of our patients who had undergone a cup revision the surgeons reported significant PE wear perioperatively. The liner used in the current study is not “cross-linked” and this, along with the head migration observed in the non-revised eight hips, indicates poor liner quality.

### Strengths and limitations

The strengths of our study are the prospective design, the complete follow-up of all patients on implant survival and DXA and X-ray mid-term clinical follow-up. As far as we know, no other prospective studies on the TOP cup with eight-year follow-up exist. Moreover, we are unaware of any other study on uncemented cups with eight-year DXA follow-up for pBMD.

Our study also has limitations. The number of patients is limited, with only 23 available for clinical follow-up. Still, this is the only study on an uncemented cup with extended follow-up with measurements of pBMD using high accuracy of DXA. Another limitation is the clinical assessment with the HHS. The ceiling effects of the HHS are well known, and other tools might provide a more sensitive measure to evaluate patients after a primary THA. However, we applied the same scoring method as in the original study for comparative results. In the two-year follow-up the stability of the cup was investigated using radiostereometric analysis (RSA). Unfortunately, for technical reasons, RSA could only be performed in eight patients at the eight-year follow-up, which is not enough to draw any conclusions about the stability of the cup in the long run. Nevertheless, we performed RSA analysis on the remaining eight patients. We found a mean cranial translation of 0.39 mm and a mean anterior tilt of 0.73° at eight years (see supplementary data). One randomized clinical trial (RCT) on two different uncemented cups reported a mean proximal translation of 0.05 mm and 0.21 mm respectively, at five years [28], while another RCT on an uncemented dual mobility cup reported a proximal translation of 0.21 mm at six years [29]. However, we did not see any signs of cup migration on plain radiography, although this technique has limitations compared to examination with RSA. In the future, the development of CT-based imaging technologies such as CT-based micromotion analysis, could overcome the current technical problems with RSA [30]. Unfortunately, RSA analysis could not be performed at two and eight years for the four patients with aseptic loosening. Thus, RSA could not provide guidance as to the cause of cup loosening for these four patients. The original study was designed using such inclusion criteria, which restricts the results from being generalized to other patient groups. On the other hand, in clinical praxis most of the patients receiving uncemented hip arthroplasty are the same as those investigated in our study.

## Conclusions

Inferior survival rates at the eight-year follow-up were seen for the TOP cup. Periprosthetic BMD in the proximal zones continues to decline eight years after surgery, indicating that this type of uncemented cup cannot prevent periprosthetic bone loss. The etiology of the periprosthetic demineralization seen in our study is a combination of mechanisms such as backside wear, stress shielding, biological reaction to wear debris and maybe poor early implant stability.

## Electronic supplementary material

Below is the link to the electronic supplementary material.

Supplementary Material 1

## Data Availability

The datasets used and/or analyzed during the current study are available from the corresponding author on reasonable request.

## Abbreviations

CI: Confidence interval
CT: Computed Tomography
DXA: Dual-energy X-ray absorptiometry
HA: Hydroxyapatite
HHS: Harris Hip Score
KM: Kaplan-Meier
pBMD: Periprosthetic bone mineral density
PE: Polyethylene
RSA: Radiostereometric analysis
THA: Total hip arthroplasty
TOP: Trabeculae-oriented pattern
UHMWPE: Ultra-high-molecular-weight polyethylene
