# Supplementary material for: Continuous periprosthetic bone loss around the TOP® cup and inferior survival rate at an 8-year follow-up: a prospective cohort study
Source: BMC Musculoskelet Disord. 2024 Sep 16;25:741. doi: 10.1186/s12891-024-07865-5 (PMC11403771; doi:10.1186/s12891-024-07865-5)
Supplement: Supplementary file 1 — Supplementary Material 1 [file 12891_2024_7865_MOESM1_ESM.docx]

|  |  | Cup migration 0 - 8 years | | Cup migration 2 - 8 years | |
| --- | --- | --- | --- | --- | --- |
|  | N | Mean  (95% CI) | Range | Mean  (95% CI) | Range |
| X-transalation (mm) medial+/lateral- | 8 | -0.07  (-0.24 – 0.1) | -0.27 − 0.39 | -0.09  (-0.17 to -0.01) | -0.23 − 0.03 |
| Y-translation (mm) proximal+/distal- | 8 | 0.39  (0.14 – 0.64) | 0.01 − 0.78 | 0.09  (0.01 – 0.16) | -0.03 − 0.22 |
| Z-translation (mm) anterior+/posterior- | 8 | 0.26  (0.13 – 0.39) | 0.06 − 0.41 | 0.13  (-0.01 – 0.26) | -0.09 − 0.34 |
| X-rotation (°) anterior tilt+/posterior tilt- | 8 | 0.73  (-0.49 – 1.95) | -1.57 − 3.32 | 0.25  (0.02 – 0.48) | -0.18 − 0.79 |
| Y-rotation (°) retroversion+/anteversion- | 8 | 0.78  (-0.10 – 1.66) | -0.54 − 2.21 | 0.38  (-0.09 – 0.84) | -0.03 −1.53 |
| Z-rotation (°) increased+/decreased- inclination | 8 | -0.17  (-0.83 – 0.49) | -1.15 − 0.83 | 0  (-0.31 – 0.31) | -0.42 − 0.5 |

Table S1: Migration of the TOP cup at 8 years, compared to baseline and 2 years, as measured with radiostereometric analysis (8 patients).


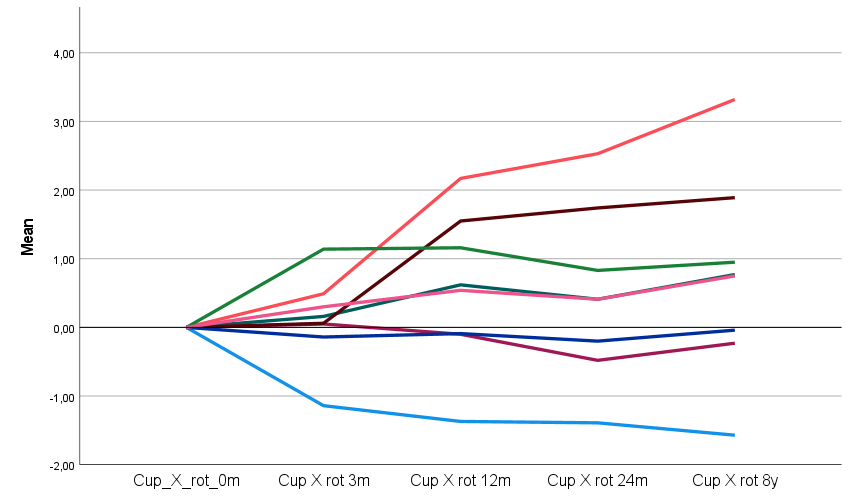

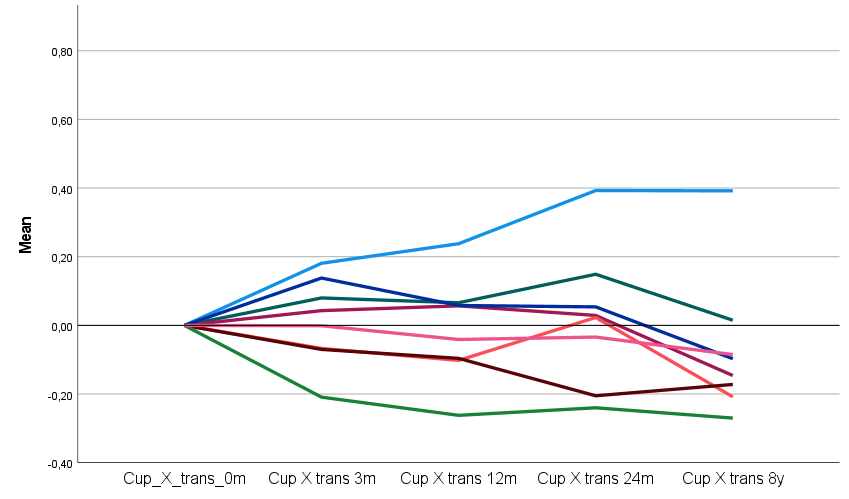

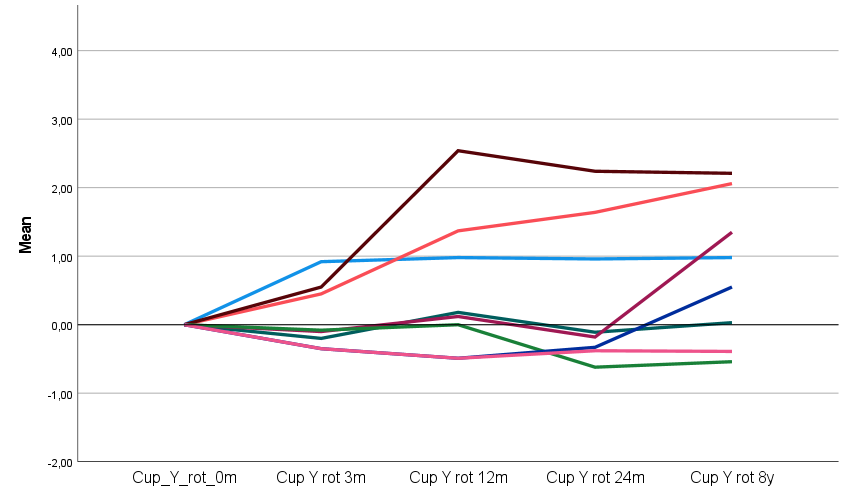

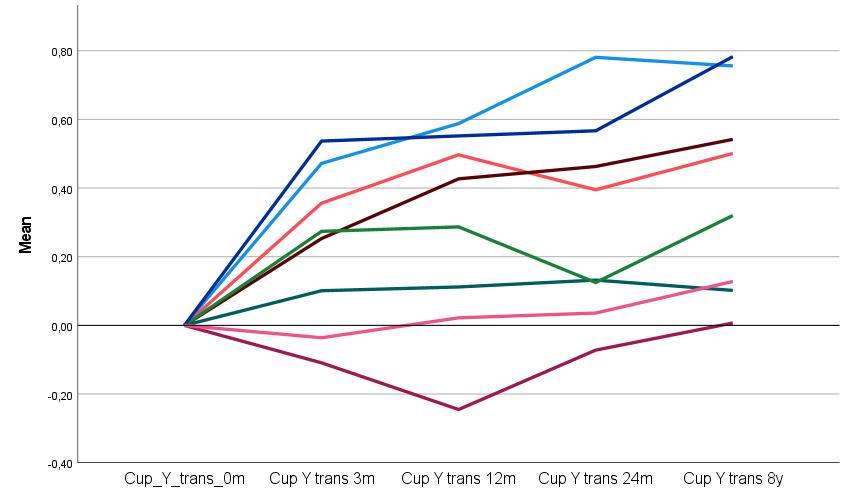

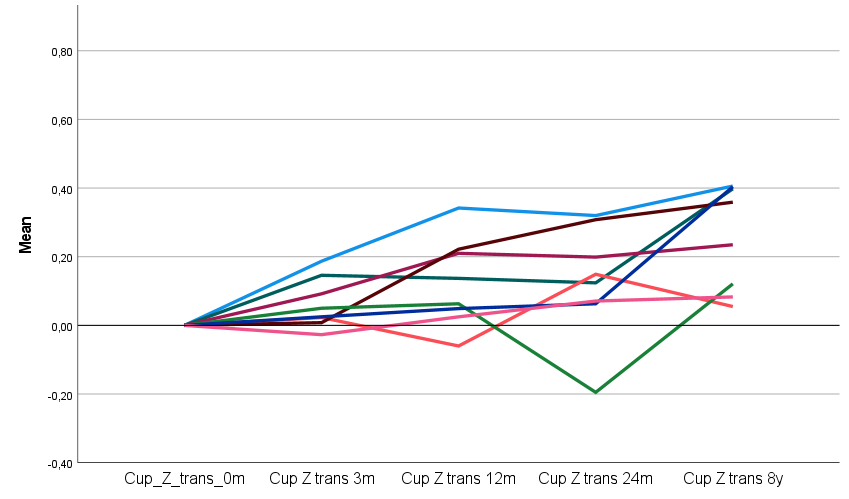

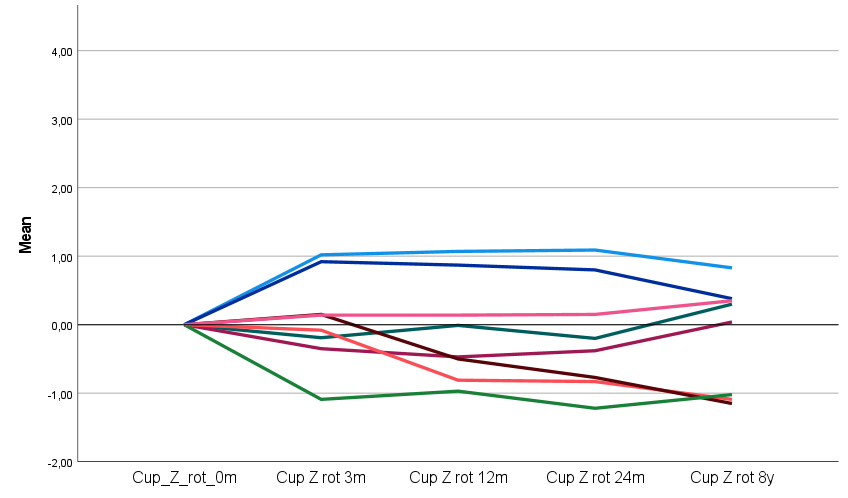


Figure S1: Migration of all individual components around and along axes in space up to 8 years at the different time points (n=8).


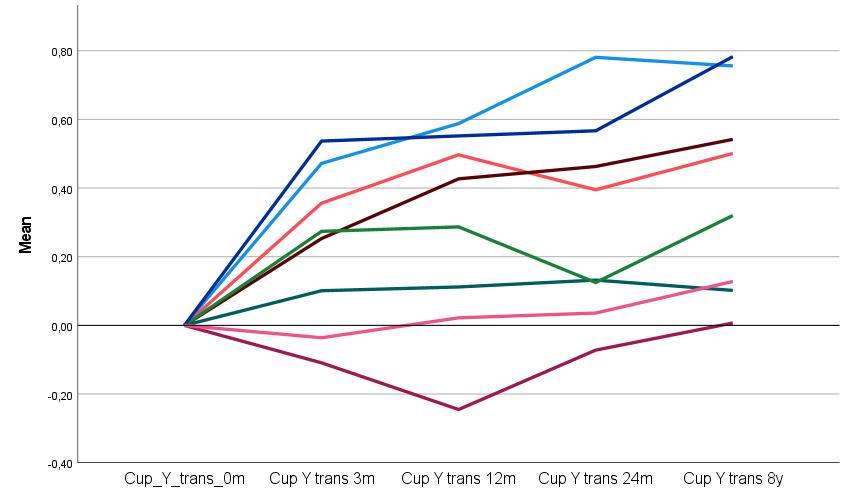


Figure S2: Translation of the cup along the y-axis up to 8 years at the different time points (n=8).
